# Competitive antagonistic action of laccase between *Trichoderma* species and the newly identified wood pathogenic *Ganoderma camelum*

**DOI:** 10.3389/fmicb.2024.1408521

**Published:** 2024-09-25

**Authors:** Aisha Umar, Mohamed S. Elshikh, Reem M. Aljowaie, Juma Mahmud Hussein, Laurent Dufossé, Chenghong Wu, Junxing Lu

**Affiliations:** ^1^Chongqing Key Laboratory of Plant Environmental Adaptations, College of Life Science, Chongqing Normal University, Chongqing, China; ^2^Institute of Botany, University of the Punjab, Lahore, Pakistan; ^3^Department of Botany and Microbiology, College of Science, King Saud University, Riyadh, Saudi Arabia; ^4^Department of Molecular Biology and Biotechnology, University of Dar es Salaam, Dar es Salaam, Tanzania; ^5^CHEMBIOPRO Laboratoire de Chimie et Biotechnologie des Produits Naturels, ESIROI Agroalimentaire, Université de La Réunion, Saint-Denis, Ile de La Réunion, France

**Keywords:** growth rate, ITS, laccase, phylogeny, wood degradation

## Abstract

*Ganoderma*, a well-known genus in the Ganodermataceae family, has caused the extinction of several tree species due to its pathogenicity. This study explored the pathogenic effect of a newly identified *Ganoderma* species on trees and its competitive efficiency against *Trichoderma* species. *Ganoderma camelum* sp. nov. is characterized by small sessile basidiomata and a velvety, soft, camel-brown pileus. Phylogenetic analysis and ITS rDNA sequences indicated that the species were *Trichoderma* and *Ganoderma camelum*. Both fungal species competed antagonistically by secreting laccase. The laccase activity of *G. camelum*, with a value of 8.3 ± 4.0 U/mL, demonstrated the highest competitive activity against *Trichoderma* species. The laccase produced by *T. atroviride* (2.62 U/mL) was most effective in countering the pathogenic action of the novel *G. camelum*. The molecular weights of laccase were determined using SDS-PAGE (62.0 kDa for *G. camelum* and 57.0 kDa for *T. atroviride*). Due to the white rot induced by this *Ganoderma* species in the host tree, *G. camelum* showed the highest percentage inhibition of radial growth (76.3%) compared to *T. atroviride* (28.7%). This study aimed to evaluate the competitive antagonistic activity of *Ganoderma* and *Trichoderma* on malt extract agar media in the context of white rot disease in the host tree. This study concluded that the laccase from *G. camelum* caused weight loss in rubber wood blocks through laccase action, indicating tissue injury in the host species. Therefore, it was also concluded that *G. camelum* was more effective in pathogenic action of the host and resisted the biological action of *T. atroviride*. In principal components analysis (PCA), all the species associated with laccase exhibited a very strong influence on the variability of the system. The PIRG rate (percentage inhibition of radial growth) was strongly and positively correlated with laccase activity.

## Introduction

*Ganoderma* is a genus widely distributed across desert, tropical, temperate, and agricultural ecosystems (Torres-Torres et al., 2015). Species within this genus exhibit diverse ecological relationships, including facultative, saprophytic, or parasitic associations with woody plants, particularly those in the Quercus-Pinus lineage (Torres-Torres et al., 2015). *Ganoderma* is a distinguished wood-decaying fungus, which affects both deceased and living trees (Pinar and Rodríguez-Couto, 2024).

These fungi also act as plant pathogens, causing basal stem rot in rubber, oil palm, and coconut trees (Kumari et al., 2024). As a basidiomycete, *Ganoderma* infects both young and mature trees, precipitating white rot through the decomposition of lignin, polysaccharides, and cellulose in hardwoods (Charpentier-Alfaro et al., 2023).

Various biotic factors, including basal rot caused by wound-colonizing fungi, contribute to the depletion of tree vegetation. In arid and semiarid regions, this disease targets the trunks, branches, and roots of woody plants (Al-Mosawi et al., 2024). Wood decay and discoloration are prevalent, and severe symptoms are observed in mature trees. This process unfolds over several years and remains imperceptible within the lifespan of the affected areas.

*Ganoderma* establishes colonization in a suitable host via the parasitic invasion of root masses and residual stumps, directly linking healthy roots with infected tissues within the soil (Shariffah-Muzaimah et al., 2015). *Ganoderma camelum* is also recognized as a wood rotter, which is believed to be an effective pathogen classified as a tree-dwelling (wood pathogenic) fungus.

The growth of *Ganoderma* is hindered by the presence of competitive antagonistic fungi, such as *Trichoderma* (Tong et al., 2020; Wang et al., 2022). *Trichoderma*, a soil-borne pathogen and an anamorphic form of *Hypocrea* (Ascomycota) (Kumar et al., 2023), induces biochemical responses in plants, thereby bolstering their defense against pathogens. *Trichoderma* Pers. is globally distributed and found in soil or decaying wood (Singh et al., 2020). A few *Trichoderma* species are renowned as industrial cellulose producers (Bischof et al., 2016) and are associated with diseases in mushrooms (Gangwar et al., 2018; An et al., 2022), including *Ganoderma lucidum* (Lu et al., 2016), *Agaricus bisporus*, *Pleurotus ostreatus*, and *Lentinula edodes* (Wang et al., 2016; Innocenti et al., 2019). These fungi are characterized by rapid growth and the production of a diverse array of degradative enzymes, e.g., laccase. Different *Trichoderma* species display antagonistic activity against other fungi through anastomosis around their hyphae, establishing a mycoparasitic relationship that curtails the activity and proliferation of pathogenic fungi affecting plants and trees (Chen et al., 2014; Yassin et al., 2021).

Investigations into the potential of laccase have encompassed various kingdoms (plants, bacteria, insects, and fungi), with fungal laccases demonstrating superior activity compared to other taxa. In higher fungi, laccase potential is particularly pronounced in its properties (Cázares-García et al., 2013; Loi et al., 2021). Fungal laccases are extensively involved in intracellular (extracellular) secretion processes, such as delignification, pathogenesis, and pigmentation (Tamano et al., 2022; Nazar et al., 2023; Rahman et al., 2024). *Ganoderma*, *Pleurotus ostreatus*, and *Trametes versicolor* are considered model organisms for laccase production (Yang et al., 2017), offering versatility and high potential for bioremediation strategies in various applications, including petrochemical, medical, textile, pesticide, and pharmaceutical waste treatment (Dong et al., 2023; Gutierrez-Rangel et al., 2024). Enzyme-assisted degradation of industrial and environmental effluents can be readily applied with multiple advantages (Al-Tohamy et al., 2023).

Despite the abundance of species, numerous regions remain unexplored, beyond the reach of researchers, and demand the undivided attention of mycologists. The species newly identified in this research was discovered in northern Pakistan, with its diminutive basidiome resembling those of the *G. lucidum* complex.

This study characterizes a novel *Ganoderma* species through morpho-anatomical, molecular, and phylogenetic analyses. The research also aimed to maximize laccase production by leveraging the competitive antagonistic interactions between *Trichoderma* and the newly identified *Ganoderma* species while also examining their wood pathogenic properties.

## Materials and methods

### Morpho-anatomical characterization, DNA extraction, PCR amplification, and phylogenetic analyses

Specimens of *Ganoderma camelum* were collected from Khanaspur Halipad, Abbottabad District, Khyber Pakhtunkhwa Province, Pakistan, between June 2018 and August 2019 and deposited under deposition numbers SCUF517 and SCUF518.

The morphological characterization of the fungus, including its color, shape, and other detailed features, were conducted according to the guidelines established by Corner (1983).

The thickness of the pileus was measured at the point where the width and length of the fruiting body intersect. The color of the pileus was determined using the color chart of Munsell (1975).

Microscopic analysis was executed by observing cross sections of the dried basidiomata, which were first soaked in a 5% potassium hydroxide (KOH) solution, then stained 1% Congo red, and subsequently visualized under a MX4300H compound light microscope (Meiji Techo Co., Ltd., Japan). At least 30 measurements were meticulously recorded at a magnification of 100X. For the spore measurements, 30 counts from two samples were presented as length × width (Nagy et al., 2010), with the apiculus excluded when not compressed. The microscopic characteristics were described in alignment with the methodology outlined by Cabarroi-Hernández et al. (2019).

Genomic DNA was isolated from the specimens using a modified CTAB technique, the ITS regions were analyzed and amplified using ITS1 and ITS2 primers (White et al., 1990). The PCR amplification process was performed within a 25 μL reaction volume, utilizing a master mix [DreamTaqGreen PCR Master Mix (2X), Fermentas].

The reaction mixture included 9.5 μL distilled water, 1 μL template DNA, 12.5 μL 2X PCR master mix, and 1 μL of each primer. The amplification protocol comprised 35 cycles of 95°C for 30 s, 52°C for 30 s, and 72°C for 1 min, concluding with a final extension of 10 min at 72°C. The PCR products were purified and sequenced by TSINGKE Co., Ltd. (China).

The dataset included DNA sequences of the novel species, along with additional ITS sequences obtained from GenBankwww.ncbi.nlm.nih.gov/genbank/ and relevant literature. The sequences were automatically aligned using MAFFT and manually adjusted using CLUSTALW in BioEdit software. A phylogenetic tree was constructed using MEGA ver. 10 and RAxML (Hall, 1999; Katoh et al., 2019). The reliability of the tree was assessed through bootstrapping with 1,000 replicates.

### Isolation and culturing

*Ganoderma camelum* was cultured by inoculating 1 cm sterile tissue segments onto Basidiomycete-selective malt extract agar (BSMEA). The BSMEA medium was prepared following the manufacturer’s guidelines (MEA) (Difco Laboratories, Franklin Lakes, NJ), augmented with streptomycin (100 mg/L) and benomyl 95% (4 mg/L) (Loyd et al., 2018b).

Terricolous fungi were isolated using the dilution plate technique with MEA, which was supplemented with Rose Bengal (1/15,000) and chloramphenicol (50 ppm) to inhibit bacterial growth (Smith and Dawson, 1944). Post-inoculation, the plates were incubated at 27°C for 10 days. Subsequently, the emerging colonies of *Trichoderma* were identified and enumerated. The samples of *Ganoderma* under study were subsequently preserved in the Suez Canal University Fungarium (SCUF) under the deposit numbers SCUF517 and SCUF518, respectively (https://ccinfo.wdcm.org/details?regnum=1180, accessed on 12 August 2024).

### Qualitative assay of laccase

The production of laccase was assessed through the placement of 5 mm-diameter disks from 7-day-old colonies onto guaiacol-supplemented agar plates (Abdel-Azeem and Salem, 2012) and by direct inoculation into modified Czapek’s agar plates. These plates were then incubated in the dark at 28°C for 7 days. The development of a pronounced brown coloration beneath and around the fungal colony was interpreted as a positive response, indicative of guaiacol oxidation (Kalra et al., 2013).

### Laccase production in submerged culture

Actively growing mycelia (five pieces of 5 mm diameter) were cultivated in 100 mL of basal nutritional medium (Umar et al., 2023) in an Erlenmeyer flask (250 mL) at 28°C and 150 rpm for 8 days from freshly prepared pure cultures of *Ganoderma* and *Trichoderma* (approximately 7 days incubation at 30°C). After incubation, the fungal broth culture containing mycelia was centrifuged at 10,000 rpm at 4°C for 20 min and then filtered using Whatman filter papers. The resulting extracellular fluid supernatant contained crude laccase, which was used for further research.

### Quantitative assay of laccase

The supernatant, containing crude laccase, was employed to assess enzymatic activity by measuring the oxidation of the guaiacol substrate (Gao et al., 2011). For this quantification, a 50 mM sodium acetate buffer, adjusted to a pH of 4.5, was combined with 2 mM guaiacol. A solution comprising 1.5 mL of the crude enzyme supernatant, 1 mL of the sodium acetate buffer, and 1 mL of guaiacol was vigorously mixed for 30 s and then incubated at 30°C for 10 min (Chefetz et al., 1998). After incubation, absorbance was measured at 465 nm (465 = 12,100 M^−1^ cm^−1^). EA = (A* V)/(t * €* v), where E.A = enzyme activity (U/mL), A = absorbance at 465 nm, V = total volume of the reaction mixture (mL), v = enzyme volume (mL), t = incubation time (min), and € = extinction coefficient ( M^−1^ cm^−1^) (Gao et al., 2011).

### Laccase purification and gel electrophoresis

The method described by Chefetz et al. (1998) was employed to purify the laccase. The filtrate was centrifuged at 13,000 rpm for 20 min at 10°C, after which the supernatant was precipitated with ammonium sulfate. The resulting precipitates were then dialyzed and loaded onto a DEAE-Cellulose anion-exchange column, which had been equilibrated with a 10 mM sodium acetate buffer (pH 5.5).

Subsequently, the laccase fraction was collected, concentrated, and dialyzed overnight, after which 3 mL of the DEAE-purified sample was applied to the column. Post-dialysis, the purity and molecular weights of the laccase were evaluated through SDS-PAGE analysis (Durán et al., 2002) and visualized using Coomassie Brilliant Blue R-250 staining. The relative molecular mass was estimated by comparison with standard molecular weight markers.

### *Ganoderma* wood decay test

The *Ganoderma* wood degradation was evaluated using rubber wood blocks, each measuring 80 mm × 50 mm × 20 mm and weighing precisely (100 g). Initially, these blocks were immersed in distilled water overnight within plastic bags and subsequently subjected to autoclaving for 45 min at a temperature of 121°C. Subsequently, 100 mL of MEA broth was administered to each block, which was then autoclaved again under identical conditions. Following a 2 min cooling period within a laminar flow hood to facilitate the adequate absorption of the medium, *Ganoderma* sp. cultures derived from Petri plates were finely minced and introduced into sterile plastic bags. An additional 100 mL of MEA was added to the blocks, which were then incubated at ambient temperature for 120 days (Loyd et al., 2018b).

Control blocks without *Ganoderma* were included. The weight of both control wood blocks and the *Ganoderma*-infected wood blocks was meticulously recorded every 20 days to ascertain the degree of decay inflicted by the *Ganoderma* species.

### Dual culture tests and percentage inhibition of radial growth

This experiment investigated the antagonistic relationship between *Ganoderma* and *Trichoderma* species. A 5 mm mycelium disk, each sourced from the periphery of an actively proliferating mycelium culture of both species, was excised and transferred to a distinct agar Petri plate. This disk was then permitted to propagate for 3 days at a controlled temperature of 25°C.

Control plates contained only *Ganoderma* cultures, and the study was replicated three times. The percentage inhibition of radial growth (PIRG) zones for both species was computed daily over 10 days, utilizing the formula delineated by Yazid et al. (2023).


$$\mathrm{PIRG}=R_{1}–R_{2}/R_{1}\times 100$$

In this formula, PIRG indicated “percentage inhibition of radial growth,” R_1_ indicated radial growth of the *Ganoderma* colony in the absence of *Trichoderma,* whereas R_2_ showed the radial growth of the *Ganoderma* colony in the presence of *Trichoderma*.

### Slide culture method

A sterile, clean glass slide was inserted into 9-cm-diameter plates to facilitate the *Ganoderma*-*Trichoderma* interaction. An autoclaved, molten MEA layer was then applied onto the slide. The 5 mm disks, excised from 1-week-old colonies on the periphery of *Ganoderma* and *Trichoderma* isolates, were positioned 3 cm apart on the MEA surface, opposite each other across the slide. To mitigate drying, a small quantity of double-distilled water was added to the plate. Subsequently, the plate was incubated at 25°C for a week. Upon the completion of the incubation, the area where *Ganoderma*-*Trichoderma* hyphae interfaced was stained with lactophenol in cotton blue, and the slide was examined under a light microscope to assess any signs of mycelial penetration and cell wall degradation that occurred during the incubation period.

### Statistical analysis

The mean values and standard deviations (±SD) from three biological replicates (*n* = 3) were presented in the data. These triplicate data sets transformed and were subsequently subjected to an ANOVA analysis using SPSS software. The mean differences were evaluated through the HSD (Tukey’s standardized range) test, with statistical significance set at Pd ≤ 0.05.

Principal component analysis (PCA) was employed to elucidate the relationships among the investigated cases and parameters. The statistical analyses were conducted using Statistica software (version 12.0, StatSoft Inc., Tulsa, OK, United States). Principal components analysis (PCA), ANOVA, and correlation determination were all performed at a significance level of *a* = 0.05. The data matrix utilized for the PCA statistical analysis of the chromatographic test results comprised three columns and 11 rows. The input matrix was automatically scaled.

## Results

### Molecular identification of *Trichoderma* and *Ganoderma*

**Figure 1:**
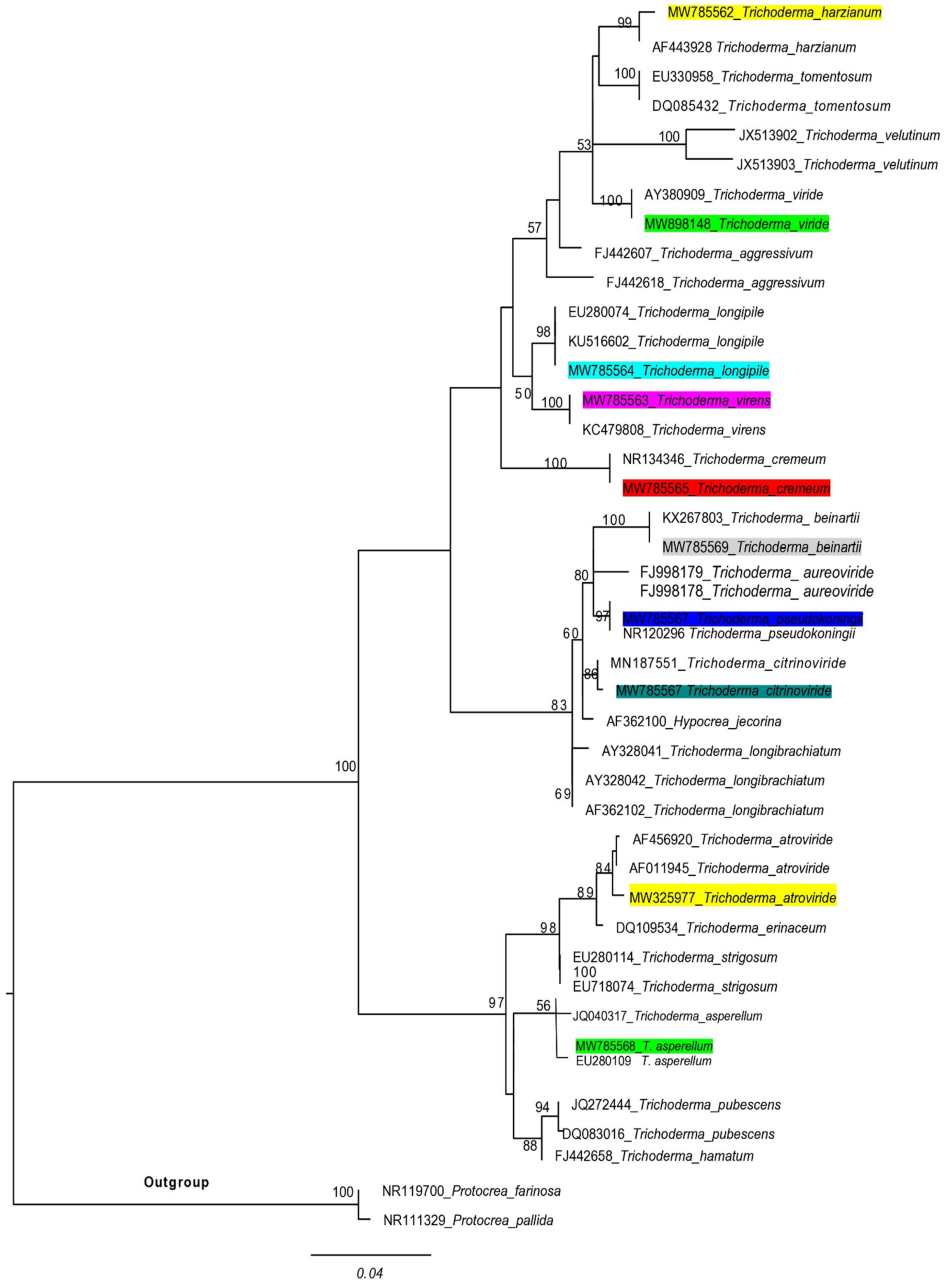
A phylogenetic tree of *Trichoderma* species was used in this experiment, and related taxa were based on ITS sequences generated by the maximum likelihood method. The tree was rooted using two species of *Protocrea farinosa* and *Protocrea pallida*. Bootstrap values (>50%) are shown at the branches.

**Figure 2:**
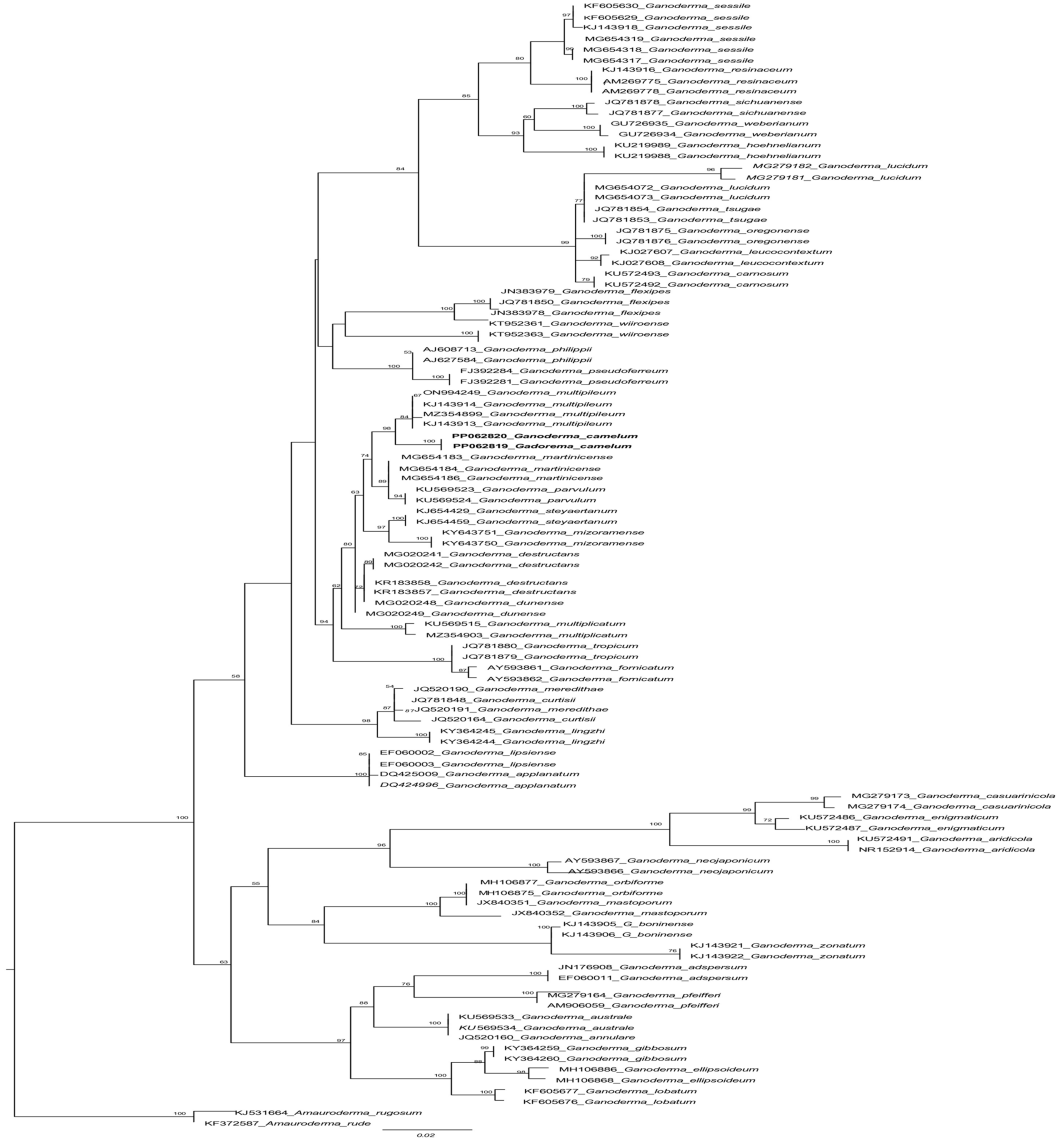
The phylogenetic tree of *Ganoderma camelum* used in this experiment and related taxa based on ITS sequences generated by the maximum likelihood method. The tree was rooted using two species from *Amauroderma* (*Amauroderma* rugosum and *Amauroderma rude*). Bootstrap values (>50%) are shown at the branches.

ITS markers were employed to ascertain the *Trichoderma* species responsible for the highest laccase production. The potential species were identified by constructing a phylogenetic tree, which was generated using maximum likelihood analysis. The purified fungal mycelium was found to cluster into a distinct clade, closely related to other species, with high bootstrap values confirming the robustness of the tree’s topology (Table 1). Phylogenetic analysis facilitated the clear identification of the filamentous *Trichoderma* species (Figure 1). Multiple sequence alignments were executed with CLUSTALW in BioEdit software, followed by manual adjustment. Subsequently, phylogenetic trees were constructed from these alignments using MEGA Version 10.0 and RAxML, with the statistical significance of the tree being evaluated through bootstrapping with 1,000 replicates. The maximum likelihood tree topology for *Ganoderma* is depicted, exhibiting a 98% statistical bootstrap value, which supports the identification of a novel species (Figure 2; Table 2).

**Table 1:** *Trichoderma* species are used in phylogenetic analyses and are representative of each species used in this study.

Taxon	GenBank No.	Country	Voucher/Strain/Isolate	References
*Trichoderma harzianum*	AF443928	Mexico	G.J.S. 00-21	Chaverri et al. (2003)
	MW785562	Pakistan	TH101	Umar (2021)
*Trichoderma tomentosum*	EU330958	Canada	DAOM 178713A	Degenkolb et al. (2008)
	DQ085432	Canada	DAOM 178713A	Samuels (2004)
*Trichoderma velutinum*	JX513903	India	IIc2a	Prashantha et al. (2024)
	JX513902	India	IIA3b	Prashantha et al. (2024)
*Trichoderma viride*	AY380909	United States	ATCC 28038	Holmes et al. (2004)
	MW898148	Pakistan	IAGST22	This study
*Trichoderma aggressivum*	FJ442607	Ireland	CBS 100526	Chaverri et al. (2003)
	FJ442618	Ecuador	DIS 252E	Chaverri et al. (2003)
*Trichoderma longipile*	EU280074	Canada	DAOM 1772271a	Hoyos-Carvajal et al. (2009)
	KU516602	Poland	75Jb14	Jankowiak et al. (2016)
	MW785564	Pakistan	TL103	This study
*Trichoderma virens*	MW785563	Pakistan	TV102	Umar (2021)
	KC479808	Indonesia	GL2	Shittu et al. (2017)
*Trichoderma cremeum*	NR 134346	United States	BPI 1112894	Samuels (2004)
	MW785565	Pakistan	TC104	This study
*Trichoderma beinartii*	KX267803	South Africa	PPRI 19281	du Plessis et al. (2018)
	MW785569	Pakistan	TB109	This study
*Trichoderma aureoviride*	FJ998179	China	FJD22	Xia et al. (2011)
	FJ998178	China	FJD2	Xia et al. (2011)
*Trichoderma pseudokoningii*	MW785566	Pakistan	TL105	This study
	NR 120296	United States	NS19	Kuhls et al. (1996)
*Trichoderma citrinoviride*	MN187551	Poland	Tc19-18Ig	Baturo-Cieśniewska et al. (2020)
	MW785567	Pakistan	TC106	This study
*Hypocrea jecorina*	AF362100	Korea	KACC40517	Park et al. (2005)
*Trichoderma longibrachiatum*	AY328041	Hungary	UAMH 7956	Szekeres et al. (2006)
	AY328042	Hungary	ATCC 208859	Szekeres et al. (2006)
	AF362102	Korea	T9	Park et al. (2005)
*Trichoderma atroviride*	AF456920	United States	DAOM 222096	Dodd et al. (2003)
	AF011945	Austria	UAMH 7956	Kindermann et al. (1998)
	MW325977	Pakistan	TAPU_07	Umar (2021)
*Trichoderma erinaceum*	DQ109534	Peru	DIS 7	Samuels et al. (2006)
*Trichoderma strigosum*	EU280114	Guatemala	DAOM 234231	Hoyos-Carvajal et al. (2009)
	EU718074	Germany	DMC 787b	Douanla-Meli et al. (2013)
*Trichoderma asperellum*	JQ040317	China	HNZZ1006	Sun et al. (2012)
	MW785568	Pakistan	TAS107	Umar (2021)
	EU280109	Colombia	CIB T05	Hoyos-Carvajal et al. (2009)
*Trichoderma pubescens*	JQ272444	United States	16B5	Baird et al. (2014)
	DQ083016	United States	DAOM 166162	Samuels (2004)
*Trichoderma hamatum*	FJ442658	Ecuador	DIS 358H	Chaverri et al. (2003)
*Protocrea farinosa*	NR119700	Austria	CBS 121551	Jaklitsch et al. (2008)
*Protocrea pallida*	NR111329	Austria	CBS 299.78	Jaklitsch et al. (2008)

**Table 2:** Species used for phylogenetic analyses of this study and their corresponding GenBank accession numbers.

Taxon	Accession no.	Origin	Voucher/Strain/Isolate	References
*Ganoderma sessile*	KF605630	United States	JV 1209/27	Zhou et al. (2015)
	KF605629	United States	JV 1209/9	Zhou et al. (2015)
	KJ143918	United States	NY 00985711	Zhou et al. (2015)
	MG654319	United States	228 DC	Loyd et al. (2018a)
	MG654318	United States	210FL	Loyd et al. (2018a)
	MG654317	United States	200MO	Loyd et al. (2018a)
*Ganoderma resinaceum*	KJ143916	Netherlands	CBS 194.76	Loyd et al. (2018a)
	AM269775	Italy	DP1	Guglielmo et al. (2007)
	AM269778	Italy	G4/13	Guglielmo et al. (2007)
*Ganoderma sichuanense*	JQ781878	China	Cui 7691 (BJFC)	Zhou et al. (2015)
	JQ781877	China	HMAS 42798	Zhou et al. (2015)
*Ganoderma weberianum*	GU726935	India	GW-11	Mohanty et al. (2011)
	GU726934	India	GW-10	Mohanty et al. (2011)
*Ganoderma hoehnelianum*	KU219989	China	Dai 12096	Song et al. (2016)
	KU219988	China	Dai11995	Song et al. (2016)
*Ganoderma lucidum*	MG279182	China	Cui 14405	Xing et al. (2018)
	MG279181	China	Cui 14404	Xing et al. (2018)
	MG654072	United States	UMNUT8	Loyd et al. (2018a)
	MG654073	United States	UMNUT9	Loyd et al. (2018a)
*Ganoderma tsugae*	JQ781854	China	Yuan5649	Loyd et al. (2018a)
	JQ781853	China	Dai3937	Loyd et al. (2018a)
*Ganoderma oregonense*	JQ781875	United States	CBS 265.88	Zhou et al. (2015)
	JQ781876	United States	CBS 266.88	Zhou et al. (2015)
*Ganoderma leucocontextum*	KJ027607	China	GDGM443 03	Li and Yuan (2015)
	KJ027608	China	GDGM44304	Li and Yuan (2015)
*Ganoderma carnosum*	KU572493	Czech R	JV 8709/8	Du et al. (2023)
	KU572492	Czech R	MJ 21/08	Du et al. (2023)
*Ganoderma flexipes*	JN383979	China	Wei 5494 (IFP)	Zhou et al. (2015)
	JN383978	China	Wei5200	Zhou et al. (2015)
	JQ781850	China	Wei5491	Cao et al. (2012)
*Ganoderma wiiroense*	KT952361	Ghana	UMN-20-GHA	Crous et al. (2015)
	KT952363	Ghana	UMN-21-GHA	Crous et al. (2015)
*Ganoderma philippii*	AJ608713	Malaysia	E7425	Hapuarachchi et al. (2018)
	AJ627584	Malaysia	FRIM 589	Hapuarachchi et al. (2018)
*Ganoderma pseudoferreum*	FJ392284	China	CATASGp008	Costa-Rezende et al. (2017)
	FJ392281	China	CATASGp005	Costa-Rezende et al. (2017)
*Ganoderma multipileum*	ON994249	China	HKAS 123775	He et al. (2022)
	KJ143914	China	Dai 9447	Zhou et al. (2015)
	MZ354899	China	Cui 13597	Sun et al. (2022)
	KJ143913	China	CWN 04670	Zhou et al. (2015)
*Ganoderma camelum*	PP062819	Pakistan	SCUF517	This study
	PP062820	Pakistan	SCUF518	This study
*Ganoderma martinicense*	MG654184	United States	235TX	Loyd et al. (2018a)
	MG654183	United States	232GA	Loyd et al. (2018a)
	MG654186	United States	248NC	Loyd et al. (2018a)
*Ganoderma parvulum*	KU569523	Colombia	CC16	Bolaños et al. (2016)
	KU569524	Colombia	CC17	Bolaños et al. (2016)
*Ganoderma steyaertanum*	KJ654429	Indonesia	IV-54-3	Glen et al. (2014)
	KJ654459	Indonesia	6-WN-15-M-A	Glen et al. (2014)
*Ganoderma mizoramense*	KY643751	India	UMN-MZ4	Crous et al. (2017)
	KY643750	India	UMN-MZ5	Crous et al. (2017)
*Ganoderma destructans*	MG020241	South Africa	CMW42140	Tchotet Tchoumi et al. (2018)
	MG020242	South Africa	CMW42141	Tchotet Tchoumi et al. (2018)
	KR183858	South Africa	CMW43672	Coetzee et al. (2015)
	KR183857	South Africa	CMW4367 1	Coetzee et al. (2015)
*Ganoderma dunense*	MG020248	South Africa	CMW42149	Du et al. (2023)
	MG020249	South Africa	CMW 42150	Du et al. (2023)
*Ganoderma multiplicatum*	KU569515	Colombia	CC8	Bolaños et al. (2016)
	MZ354903	China	Dai 17395	Sun et al. (2022)
*Ganoderma tropicum*	JQ781880	China	Yuan 3490 (IFP)	Zhou et al. (2015)
	JQ781879	China	Dai 9724 (IFP)	Zhou et al. (2015)
*Ganoderma fornicatum*	AY593861	China	AS5.539	Wang and Yao (2005)
	AY593862	China	AS5.538	Wang and Yao (2005)
*Ganoderma meredithae*	JQ520190	Korea	ATCC 64492	Park et al. (2012)
	JQ520191	Korea	ASI 7140	Park et al. (2012)
*Ganoderma curtisii*	JQ781848	Korea	CBS 100131	Park et al. (2012)
	JQ520164	Korea	CBS 100132	Park et al. (2012)
*Ganoderma lingzhi*	KY364245	South Korea	SFC20150624-06	Jargalmaa et al. (2017)
	KY364244	South Korea	SFC20120721-08	Jargalmaa et al. (2017)
	KY364245	South Korea	SFC20150624-06	Jargalmaa et al. (2017)
*Ganoderma lipsiense*	EF060002	Finland	NOR74/67/5	Terho et al. (2007)
	EF060003	Finland	FIN R330 2la	Terho et al. (2007)
*Ganoderma applanatum*	DQ425009	China	GA165	Costa-Rezende et al. (2017)
	DQ424996	China	GA117	Costa-Rezende et al. (2017)
*Ganoderma casuarinicola*	MG279173	China	Dai 16336	Du et al. (2023)
	MG279174	China	Dai 16337	Du et al. (2023)
*Ganoderma enigmaticum*	KU572486	South Africa	Dai 15970	Du et al. (2023)
	KU572487	South Africa	Dai 15971	Du et al. (2023)
*Ganoderma aridicola*	KU572491	South Africa	Dai 12588	Du et al. (2023)
	NR152914	South Africa	BJFC Dai 12588	Xing et al. (2016)
*Ganoderma neojaponicum*	AY593867	China	AS5.542	Wang and Yao (2005)
	AY593866	China	AS5.541	Wang and Yao (2005)
*Ganoderma orbiforme*	MH106877	China	GACP1408 1329	Hapuarachchi et al. (2018)
	MH106875	China	GACP1408 1235	Hapuarachchi et al. (2018)
*Ganoderma mastoporum*	JX840351	China	TNMF0018835	Wang et al. (2022)
	JX840352	China	TNM-F0018783	Wang et al. (2022)
*Ganoderma boninense*	KJ143905	Japan	WD 2028 (FFPRI)	Zhou et al. (2015)
	KJ143906	Japan	WD 2085	Zhou et al. (2015)
*Ganoderma zonatum*	KJ143921	United States	FL-02 (TNM)	Zhou et al. (2015)
	KJ143922	United States	FL-03 (TNM)	Zhou et al. (2015)
*Ganoderma adspersum*	JN176908	Italy	PF263	Hapuarachchi et al. (2018)
	EF060011	Finland	ITA 39	Terho et al. (2007)
*Ganoderma pfeifferi*	MG279164	China	Dai 12153	Xing et al. (2018)
	AM906059	Italy	PLN 22	Guglielmo et al. (2008)
*Ganoderma australe*	KU569533	Colombia	CTRA3	Bolaños et al. (2016)
	KU 569534	Colombia	CTRA4	Bolaños et al. (2016)
*Ganoderma annulare*	JQ520160	Korea	KCTC 16803	Dai et al. (2011)
*Ganoderma gibbosum*	KY364259	South Korea	SFC20130404-21	Jargalmaa et al. (2017)
	KY364260	South Korea	SFC20140702-12	Jargalmaa et al. (2017)
*Ganoderma ellipsoideum*	MH106886	China	GACP1408 1215	Hapuarachchi et al. (2018)
	MH106868	China	GACP1408 0968	Hapuarachchi et al. (2018)
*Ganoderma lobatum*	KF605677	United States	JV 0402/24	Hapuarachchi et al. (2018)
	KF605676	United States	JV 1212/10J	Hapuarachchi et al. (2018)
*Amauroderma rugosum*	KJ531664	China	Cui 9011	Li and Yuan (2015)
*Amauroderma rude*	KF372587	China	GDGM25736	Li et al. (2015)

### Morpho-anatomical description of *Trichoderma* colonies and *Ganoderma* sp.

This study comprised 10 *Trichoderma* species and one new *Ganoderma* species evaluated for laccase activity. The colony characteristics of each species were determined (Table 3).

**Table 3:** List of laccase (U/mL) producing *Ganoderma camelum* and *Trichoderma* species with PIRG and colony characteristics.

Sr No.	Species name	GenBank accessions	Conidia shape	Pigmentation	Colony appearance	PIRG rate (%)	Laccase activity (U/mL)
1	*Ganoderma camelum*	PP062819	Absent	Green	White, branched, clamped	76.3^a^	8.3 ± 4.0
2	*Trichoderma harzianum*	MW785562	Globose to Subglobose	Green	Floccose/cottony white	20^cd^	1.12 ± 0.03
3	*T. viride*	MW898148	Globose	Gray to green	Highly intricate	22.5^abcd^	1.24 ± 0.05
4	*T. pseudokoningii*	MW785566	Oblong ellipsoidal	White	Ringed	24^abc^	1.16 ± 0.01
5	*T. cremeum*	MW785565	Oblong	Creamy	Circular	25.6^abc^	1.98 ± 0.09
6	*T. longipile*	MW785564	Oblong	Incarnate/green	Circular	21^cd^	1.84 ± 0.02
7	*T. atroviride*	MW325977	Globose to subglobose	Green	Highly intricate	28.7^ab^	2.62 ± 0.01
8	*T. citrinoviride*	MW785567	Ellipsoidal	Yellowish green	Rough and pigmented	15.8^de^	1.65 ± 0.01
9	*T. beinartii*	MW785569	Smooth oblong	Diffusible	Concentric zones	20^e^	0.42 ± 0.06
10	*T. asperellum*	MW785568	Subglobose/ovoidal	Green to dark green	Concentric rings	18^de^	1.25 ± 0.08
11	*T. virens*	MW785563	Ellipsoidal to obovoid	Light yellow/green	Floccose	22^abcd^	1.03 ± 0.07

### Taxonomy

**Figure 3:**
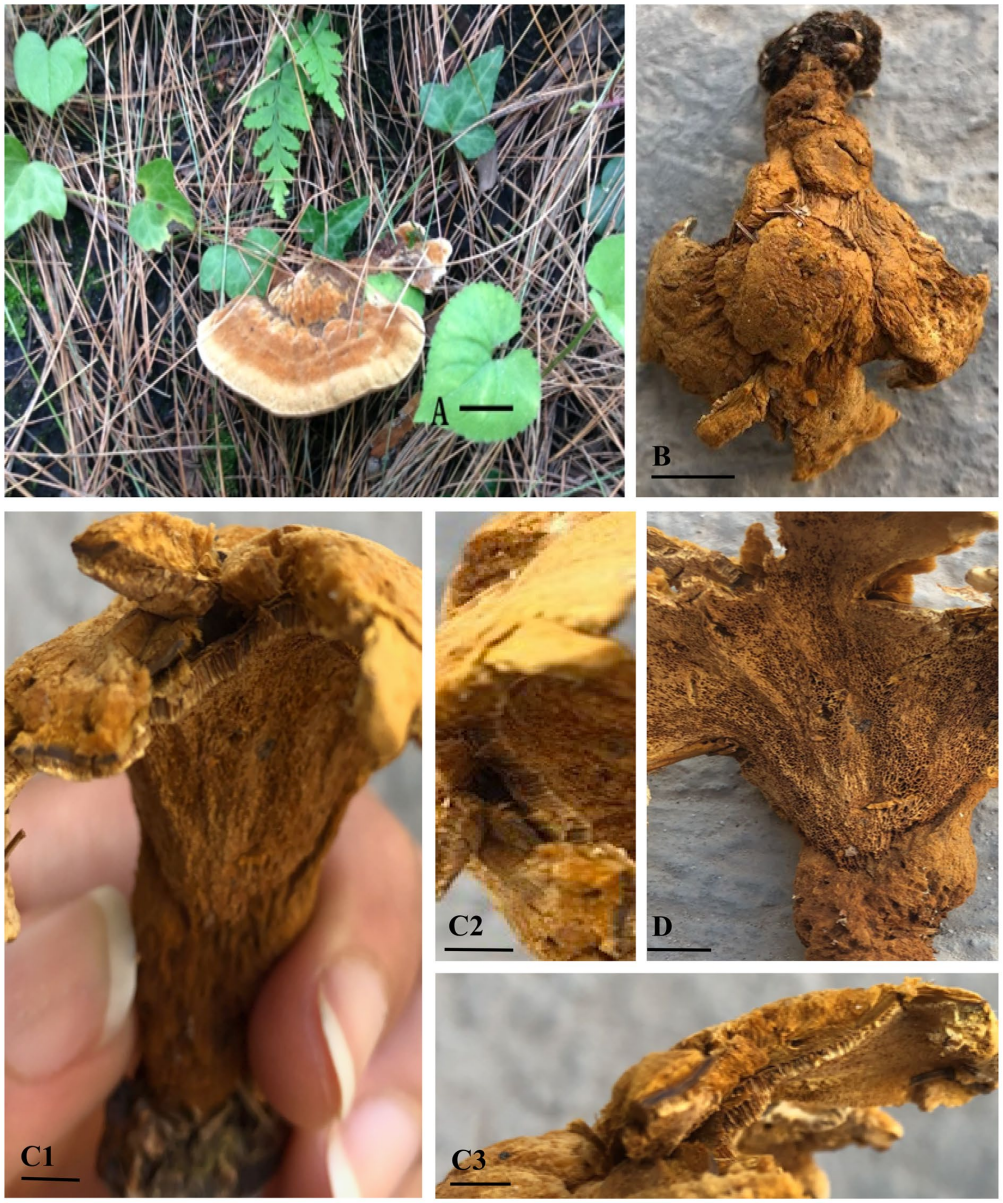
*Ganoderma camelum* **(A,B)**. Basidiome upper surface (**A**: fresh, **B**: dried). **(C1)** Contextum and Tubes. **(C2)** Tubes. **(C3)** Contextum. **(D)** Lower pore surface. (Scale bars: **A,B** = 10 mm, **C–E** = 5 mm).

**Figure 4:**
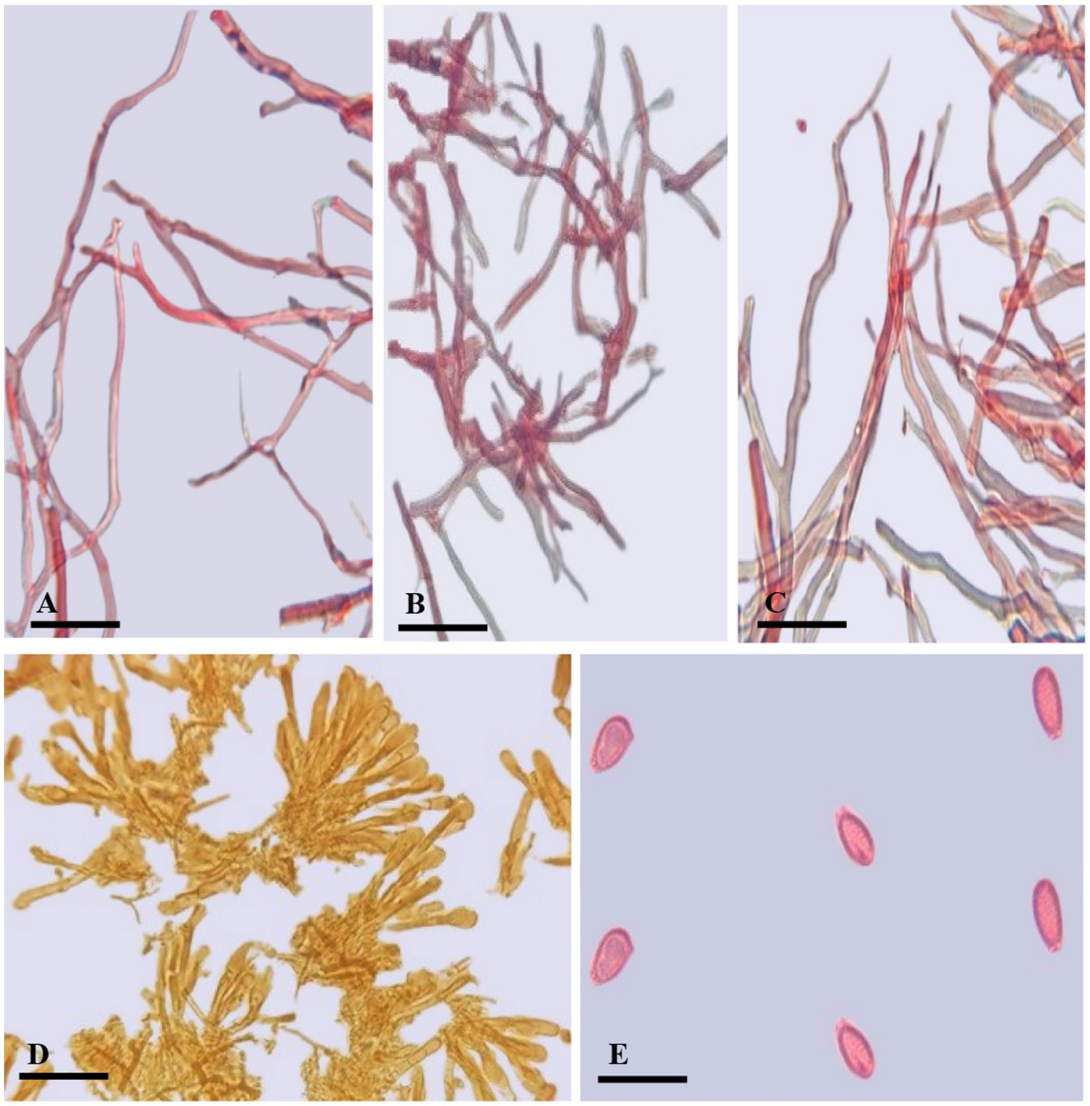
*Ganoderma camelum* **(A)** Generative hyphae. **(B)** Binding hyphae. **(C)** Skeletal hyphae. **(D)** Crustohymeniderm cells. **(E)** Basidiospores. (Scale bars: **A–C** = 5 μm, **D–E** = 10 μm).

***Ganoderma camelum*** A. Umar, sp. nov. (Figures 3A–D, 4A–E).

MycoBank# 854703.

**Diagnosis**: In the phylogenetic tree (Figure 2), *Ganoderma martinicense* and *G. multipileum* are very closely matrixed to our new species of *G. camelum*. Morphologically, *G. camelum* is characterized by its sessile, delicate, and very soft velvety appearance of basidiomata; similarly, a sessile basidiome is found in *G. martinicense,* the closest species in the phylogenetic tree. The other closest species was *G. multipileum,* which rarely exhibits this characteristic and has stipitate basidiomata. Growth zones are found in both *G. camelum* and *G. martinicense*. The contextum is dark cinnamon brown in *G. martinicense* and light-brown to brown in *G. multipileum*, while *G. camelum* exhibited a soft light brown to camel brown contextum. The spores are larger in *G. multipileum* (7.3–) 8.0–11.5 (−12.2) × (5.3–) 5.5–7.8 (−8.3) μm and *G. martinicense* (8–) 8.8–10.5 (−11.3) × (5–) 5.5–7 (−7.2) μm, while smaller in *G. camelum* (4.7–5.2 × 2.3–3.6 μm).

**Etymology**: The species epithet “*camelum*” refers to camel brown color.

**Holotype**: PAKISTAN, Khyber Pakhtunkhwa, Abbottabad District, Khanspur Halipad (34^°^ 1′ 16 N, 73^°^ 25′ 40E, on the living stem of *Pinus wallichiana,* elevation 2,250 m above sea level, Aisha Umar, 6th June 2018, KPKHP31) (SCUF517: GenBank PP062819).

**Description**: **Basidiomata** sessile, convex, velvety, very soft, shallow waved five concentric zones, inner four zones tawny olive (10YR) to camel brown (5YR), outer zone pinkish buff (9/4R) to beige (9/6R); **Margin** 0.1–0.2 mm, very thin, white; **Pileus** 5.5–6.5 × 6.5–7.3 cm, glabrous, bumpy, verrucose, reniform, thick, margins thin, obtuse, slight radiating lines, soft flesh; **Pores** 110–153 × 125–130 μm, hard porous layer, yellow-brown (5YR) to tawny (10YR), subcircular or longitudinal; **Tubes** 0.2–0.3 mm long, min, non-stratified, ochraceous buff (6/8YR); **Context** 0.4–0.5 mm thick, light brown (6/6YR) soft velvety layer, beneath brown tawny (10YR) hard layer, milky cream, dry, fibrous and corky; **Crustohymeniderm** palissade club-shaped, clavate, pale yellow to yellow (5Y), 32.5–52.7 × 10.2–12.4 μm, smooth, double-walled, few two to three septate or few multi-septate cells; **BASIDIOLES** 6.5–13 × 4.5–7 μm, inverted pear-shaped to broadly clavate with big oil droplets; **Basidiospores** 4.7–5.2 × 2.3–3.6 μm A_L_ = 4.6 μm, A_W_ = 2.8 μm, Q = 1.63 (*n* = 30/1), ellipsoid with tapering ends, smooth, bitunicate, inter-walled pillars absent, laterally pointed; **Hyphal System Trimitic** (1) generative hyphae (septate, clamped, colorless, thin-walled, 2–3.4 μm), (2) skeletal hyphae (thick-walled, colorless, unbranched or few branches with distal end, 3.1–4.8 μm), and (3) binding hyphae (arboriform, colorless thick-walled, much-branched, 1.2–3.4 μm).

**Additional specimen examined**: PAKISTAN, Khyber Pakhtunkhwa, Abbottabad District, Khanspur Halipad (34^°^ 1′ 16 N, 73^°^ 25′ 40E), on the living stem of *Pinus wallichiana,* elevation 2,215 m above sea level, Aisha Umar, 25 August 2019, KPKHP32 (SCUF518:GenBank PP062820).

### Results of the BLAST program

The consensus sequence of the ITS regions was subjected to a BLAST search utilizing the NCBI GenBank database, facilitating a comparison with a sequence database to ascertain species-level taxonomic data. An unknown or novel species is identified by situating it within an evolutionary context alongside other homologous sequences through phylogenetic analyses. The initial BLAST results for our sequences and consensus yielded 100 NCBI BLAST hits, of which over 58 were designated merely as “*Ganoderma* sp.” (merely the genus name was provided without specifying the species). These 58 sequences of *Ganoderma* species remained unnamed before our initial BLAST analysis. Consequently, this study has assigned species names to all previously unknown sequences associated with the genus *Ganoderma*. In the NCBI Query Cover, our novel species matched 100% with previously unidentified *Ganoderma* species. The initial BLAST revealed a 99.66% identification percentage, with an accuracy length of 619 (Accession PP062820.1) and 621 (Accession PP062819.1). The maximum score and total score for our new sequences were 1,081, suggesting that this represents a species not previously described.

### Comparison of *Ganoderma camelum* with neighboring species of a phylogenetic tree

In the phylogenetic tree, several neighboring species are positioned in close proximity to our newly identified species. These include *Ganoderma multipileum* (Wang et al., 2009; Welti and Courtecuisse, 2010; Nguyen et al., 2023), *G. martinicense* (Loyd et al., 2018c), *G. mizoramense* (Crous et al., 2017), and *G. parvulum* (Torres-Torres and Guzmán-Dávalos, 2012) (Figure 2). Differentiation between *G. camelum* and *G. martinicense* from *G. multipileum* is evident through the presence of sessile basidiomata in the latter, as *G. multipileum* rarely exhibits this characteristic. The basidiomes of *G. parvulum* exhibit a range from sessile to stipitate, with a more frequent stipitate form akin to *G. mizoramense*. The basidiomata of *G. mizoramense* are pileate, stipitate, applanate, flabelliform, and devoid of any “growing zones,” contrasting with *G. camelum*. The pileal surface is smooth, laccate, radially rugose, slightly zonate with dark lines, and ranges from fully reddish brown to violet brown in *G. parvulum*. In contrast, it is small, soft, non-laccate and light camel brown in our new species. The upper pileus surface of *G. mizoramense* can be distinguished from *G. camelum* by its reddish brown (fresh) to liver-brown (dried) context, dark-brownish to dark reddish brown, with a white lower surface when fresh, as opposed to the light camel brown pileus and concolorous context observed in our new species.

Concentric growth zones are present in both *G. camelum* and *G. martinicense*. The contextum is dark cinnamon brown in *G. martinicense* and light brown to brown in *G. multipileum*, while our new species exhibits a soft velvety light brown or camel brown contextum. *G. parvulum* possesses a light pale ochraceous context (Torres-Torres and Guzmán-Dávalos, 2012) with dark horny (Murrill, 1902) or carob brown (Steyaert, 1980) resinaceous streaks, differing from our new species. The context in *G. parvulum* occasionally displays scattered yellow spots and a thin yellow line just below the crust, features absent in our new species. A uniform ochraceous or cinnamon context is characteristic of *G. mizoramense*.

In *G. parvulum*, the margins are slightly lobulated, ranging from white to pale yellow or grayish-yellow to yellowish orange, contrasting with the white and non-lobulated margins of *G. camelum*. Our species features a wide yellowish-brown porous surface, contrasting the white, yellowish white, or sun yellow surface in actively growing *G. parvulum* and the yellowish brown to brownish orange surface in dried pore surfaces.

Cuticular cells are cylindrical to slightly clavate, averaging 50 μm in length, while those in our new species are palissade club-shaped, clavate, pale yellow to yellow, smooth, double-walled, with few two-to three-septate or few multi-septate cells, and smaller in size (32.5–52.7 μm × 10.2–12.4 μm).

*Ganoderma camelum* has smaller basidiospores (4.7–5.2 μm × 2.3–3.6 μm), subglobose to ellipsoid, smooth, bitunicate, and lacks laterally pointed and inter-walled pillars. In contrast, *G. parvulum* features larger spores (8.1 μm × 5.9 μm), free to subfree very thin pillars. The basidiospores of *G. mizoramense* are brown, ellipsoid with a truncate base, verruculose, and larger (11.10 μm × 7.6 μm) than those of our new species. The spore size in *G. multipileum* and *G. martinicense* is larger (7.3–) 8.0–11.5 (−12.2) × (5.3–) 5.5–7.8 (−8.3) μm and (8–)8.8–10.5(−11.3) × (5–)5.5–7(−7.2) μm, respectively, while our new species exhibits smaller spores.

### Screening of laccase-producing species

Four *Trichoderma* species, including *T. cremeum*, *T. longipile*, *T. citrinoviride*, and *T. atroviride*, were found to be capable of oxidizing guaiacol. *Ganoderma* exhibited the darkest maroon zone on the laccase detection plate, whereas *Trichoderma* demonstrated the highest laccase activity, as observed in Supplementary Figure S1A1,A2. Among the tested species, *T. atroviride* was identified as having the greatest laccase secretion potential, while *T. citrinoviride* presented the lowest. Consequently, *T. atroviride* secreted the highest amount of laccase, as depicted in Supplementary Figure S1B1,B2. On MEA media, *T. atroviride* produced pale green spores, with the prevalence of green indicating its dominant zones of operation. The growth rate within the *Ganoderma* zonal area was slower.

This study identified the most potent *Trichoderma* candidates under optimal conditions and compared their laccase activity to that of *Ganoderma camelum*, which had a laccase activity of 8.3 U/mL. *Trichoderma atroviride* was found to exhibit a laccase activity of 2.62 U/mL, establishing it as the most promising candidate for laccase production. The secondary productive species, *T. cremeum*, *T. longipile*, and *T. citrinoviride*, yielded laccase concentrations of 1.98 U/mL, 1.84 U/mL, and 1.65 U/mL, respectively (Table 3). The most robust candidate, *T. atroviride*, was selected for subsequent studies.

### Partial purification of laccase

The purification of laccase from *Trichoderma atroviride* and *Ganoderma camelum* was carried out using 60% ammonium sulfate precipitation. After partial purification, the molecular weights of the laccase were determined using SDS-PAGE. Standard protein markers were employed to estimate the purified laccase, with the band positions post-staining aiding in this quantification. The molecular weights were approximately 57.0 kDa for *T. atroviride* and 62.0 kDa for *G. camelum* (Supplementary Figure S2).

### Conflictual combat and potential of *Ganoderma*

The sequence of events commencing with reconnaissance culminates in the penetration of fungal pathogens, ultimately leading to host mortality. The secretion of laccase by *G. camelum* facilitated rapid mycelial advancement toward *Trichoderma*. In a competitive interaction, both species secreted laccase within their immediate environment, collaboratively executing the task. *Ganoderma* mycelium established physical contact with *Trichoderma* hyphae within the laccase oxidation zone, subsequently penetrating the lumen of the *Trichoderma* hyphae and assimilating its contents. Both *Ganoderma* and *Trichoderma* species released laccase enzymes or vied for space and nutrients during the antagonistic interaction.

Microscopic examination of the interaction between pathogenic *G. camelum* and *T. atroviride* revealed hyphal growth, followed by coiling, entanglement, and hooking around *G. camelum*. A change in medium color from white mycelium to purple indicated laccase secretion.

*In vitro* plate studies demonstrated that *Ganoderma* inhibited the growth of all *Trichoderma* species, which may be attributed to the higher molecular weight of laccase. An inhibition level exceeding 70%, as evaluated by the PIRG equation, was considered indicative of a species’ potential against another.

All *Trichoderma* species produced laccase at varying rates and inhibited *G. camelum* to different extents (Table 3). *Trichoderma atroviride* exhibited the highest PIRG score of 28.7%, which was statistically significant compared to other species. Regarding laccase production, five species were found to exhibit the highest protective effect against *Ganoderma*: *T. atroviride*, *T. viride*, *T. virens*, *T. pseudokoningii*, and *T. cremeum*, as determined by the PIRG evaluation.

**Figure 5:**
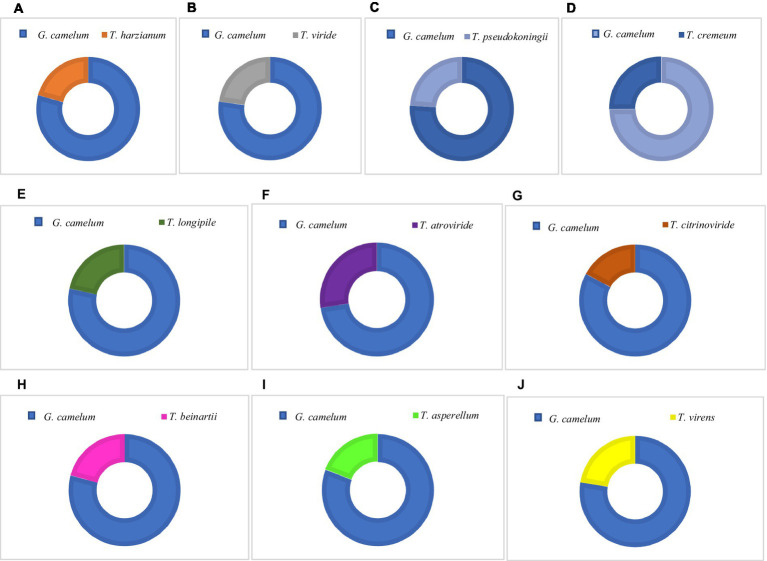
Percentage Inhibition of Radial Growth (PIRG) value of *Trichoderma* species and *G. camelum.*

Among these, *T. atroviride* secreted the highest amount of laccase, followed by *T. cremeum*, *T. longipile*, and *T. citrinoviride*. *T. atroviride* also effectively inhibited *G. camelum,* while *T. cremeum*, *T. longipile*, and *T. citrinoviride* also displayed rapid growth and purplish pigmentation after 5 days of incubation. Over a 10-day observation period, *T. citrinoviride* exhibited the smallest inhibitory zone against *G. camelum* development (76.3%) (Figures 5A–J).

The antagonistic interaction between *Ganoderma* and *Trichoderma* was confirmed through *in vitro* plate analysis, which revealed that *Ganoderma* suppressed the growth of all *Trichoderma* species. As assessed by the PIRG equation, an inhibition level above 70% was deemed indicative of a species’ antagonistic potential.

### Microscopic inhibition of mycelium

Considerable alterations in hyphal morphology were detected in *Ganoderma* mycelia upon exposure to *Trichoderma*, in contrast to the control group.

Figure 4 reveals a robust and well-developed *Ganoderma* mycelium, characterized by its healthy, compact, and highly branched structure. In response, the mycelium of *G. camelum* adopted a highly branched structure to mitigate the growth of *Trichoderma*. This adaptation was facilitated by producing a copious amount of laccase, thereby maximizing its defensive potential.

The laccase zone expanded across the entire surface of the Petri plates; however, only a limited number of hyphal strands were colonized and obscured by *Trichoderma* spores.

The hyphal structure of *T. atroviride* showed signs of disruption, aggregation, shriveling, loss, flattening, and altered appearance. Mycelial damage was attributed to intense resource competition, ultimately inhibiting *Trichoderma* growth.

The attachment of *Trichoderma* spores to the fungal hyphae indicated a mycoparasitic interaction. *Trichoderma* sp. was observed to identify and encircle adjacent fungal hyphae, forming haustoria to penetrate cell walls as a defensive strategy (Supplementary Figure S4).

### Wood decay and tree appearance

Airborne basidiospores of *G. camelum* are released through appropriate openings in the injured area of woody tissue near the ground. The basal region of the tree is prone to damage due to the moisture present in the soil.

Structural roots in the soil are damaged by basidiospores and gradually colonized, leading to tree death over several years. Structural roots anchor the tree in the soil, while fine feeder roots absorb moisture and nutrients daily.

Once structural roots are damaged, stability is at risk due to colonization by *Ganoderma* species. No effective prevention or control measures are available to overcome BSR disease until the tree’s demise. However, the *Trichoderma* species have also proved ineffective except for removing and replacing soil and trees. In this study, infected trees were compared with healthy ones as control. The vigor of *Pinus wallichiana* declined, resulting in structural weakness, slow growth, yellowing and shrinking leaves, susceptibility to wind damage, and, eventually, branch dieback.

**Figure 6:**
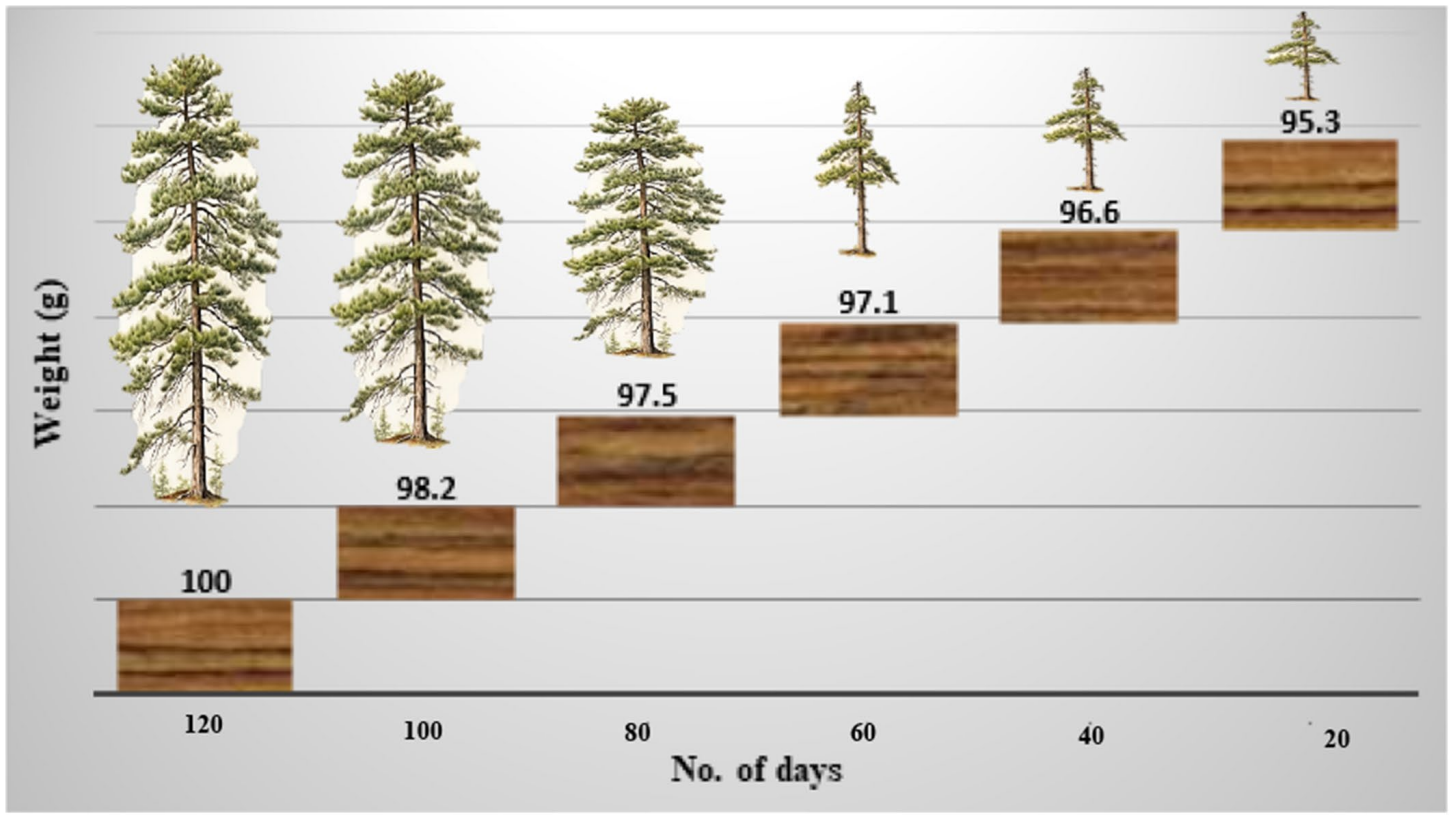
The weight of the wood block decreased over time by inoculation or infection of *Ganoderma camelum* sp. nov.

Once colonized, the wood block becomes saturated with water, resulting in a fibrous, tender, porous, crumbly, and flaky texture. The wood becomes discolored, primarily in white hues with occasional yellowish tones. The weight of the block was measured every 20 days, decreasing from 98.2 g initially to 97.5 g after 40 days, 97.1 g after 60 days, and finally 95.3 g after 120 days (Figure 6). The infected areas develop a pale, patchy appearance due to the presence of mycelium.

The woody stem becomes discolored, forming white patches due to laccase-induced degradation.

The mycelium growing within the softened wood secretes enzymes that degrade the cell wall components for energy and nourishment. Consequently, the wood undergoes whitening and bleaching due to oxidation by laccase, leading to lignin degradation.

### Principal component analysis

**Figure 7:**
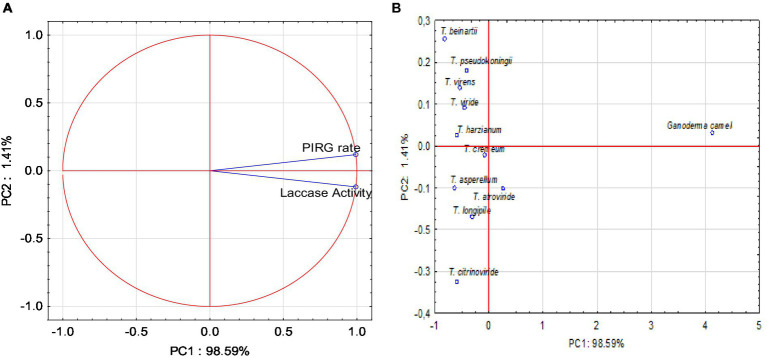
**(A)** Projection of variables: parameters on PC1 and PC2 loadings plot; **(B)** Projection of sample type on PC1 and PC2 scores plot.

Principal component analysis (PCA) was conducted, yielding two variables that accounted for 100% of the system’s variability. All parameters exerted a very strong influence on the variability of the system (Figure 7A). The PIRG rate and laccase activity parameters were strongly and positively correlated.

The PCA analysis indicated that positive values of the first principal component (PC1) described the correlation and influence of the PIRG rate and laccase parameters, accounting for 98.59% of the variance. The conducted PCA analysis demonstrated that positive values of the first main component, PC1, described the types of fungi at 98.59%. Positive values of the first principal component, PC1, disclosed the *Ganoderma* type, and negative values depicted the *Trichoderma* type. The PCA analysis (Figure 7B) also revealed that the PIRG rate and laccase parameters were described by *Ganoderma camelum*.

### Correlation matrix heat map

The heat map visually represents data, highlighting value variations through color contrasts. This graphical depiction serves as an effective means of illustrating the matrix’s values through a spectrum of colors. As detailed in Table 4, the correlation matrix shown the correlation coefficients for every pair of variables. In this study, the row and column labels consist of the “names of the variables” and the “numerical values of the calculated correlation coefficients,” which are explicitly listed within the table. The correlation coefficient, ranging from -1 to 1, indicated the strength of the linear relationship between the variables. The greater the absolute value, the more pronounced the relationship. Moreover, the sign of the correlation coefficient indicates whether the relationship between the studied variables is positive or negative.

**Table 4:** A heat map of the correlation matrix for the tested samples.

*r*>=	−1	−0.80	−0.60	−0.40	−0.20	0	0.20	0.40	0.60	0.80	1
				**PIRG rate**				**Laccase activity**			
PIRG rate				1.000				0.972			
Laccase activity				0.972				1.000			

## Discussion

*Ganoderma camelum*, a novel species discovered in Khyber Pakhtunkhwa, Abbottabad District, Khanspur Halipad, Pakistan, colonized on the stem of *Pinus wallichiana*. This species is characterized by a very soft, delicate, and velvety appearance of its basidiome, with a camel brown coloration and five distinct growth zones. The upper pileus layer is notably soft, contrasting with the slightly harder porous layer. The tubes are min, and the context is divided into two layers. The upper is soft and floccose, while the lower is flat, slightly hard, and composed. A unique feature of *Ganoderma camelum* is the presence of oval, bubble-like rounded bodies on the upper surface of its basidiome.

Phylogenetically, *Ganoderma camelum* forms a distinct clade with other *Ganoderma* species, supported by a robust statistical bootstrap value of 98%. This species is distinguished by its unique morphological characteristics and strong phylogenetic placement, thereby establishing it as a new species. *Ganoderma camelum* is differentiated from other *Ganoderma* species by its soft, delicate, and velvety nature, camel-brown coloration, and five growth zones. Its shelf-like shape further confirms its classification as a new species.

The potential for controlling BSR disease through biological means, where pathogenic *Ganoderma* species are present, has been observed. Basidiomycetes, specifically white rot fungi, degrade lignin and cellulose enzymatically, resulting in a light-colored, spongy, stringy mass that separates the firm heartwood and sapwood. White rot fungi commonly attack the hardwoods of deciduous trees, which are resistant to brown rot fungi.

Recently introduced control programs that utilize biological agents have yielded initial encouraging results in the fight against the diseases (Verma et al., 2022). The deployment of biological strategies presents a compelling alternative for managing stem rot diseases in trees, devoid of adverse environmental consequences. Unlike fungicides, biological agents colonize the rhizosphere without imparting toxic residues (Verma et al., 2023).

The integration of chemical fungicides into agricultural practices has become a fundamental aspect of crop management.

These substances, alongside pesticides, have been associated with various health issues, including cancer, respiratory ailments, and hormonal imbalances, depending on the level of exposure (Piel et al., 2019). As awareness of the detrimental impacts of excessive fungicide usage has grown, strategies for integrated pest management have been adopted to mitigate plant diseases. These approaches prioritize disease prevention and the comprehensive utilization of all available tools for plant disease management, factoring in their economic viability and toxicity. In this context, biological agents are increasingly proposed as an alternative to traditional fungicides (Ons et al., 2020). The concept of biocontrol, or biological control, often encounters confusion due to its varied interpretations in scholarly literature. Biocontrol is characterized by deploying a living organism to combat a specific plant pathogen through the secretion of diverse metabolites, antibiosis, parasitism, or competition for resources and space (Köhl et al., 2019). The use of living organisms to control plant diseases, not merely through direct antagonistic effects against plant pathogens but also via the induction of resistance, which activates the plant’s defense mechanisms, is also encompassed under the definition of a biocontrol agent (Raymaekers et al., 2020).

*Ganoderma* species are recognized for their role in wood decay, both in living trees and in decaying stumps or trunks, as documented across a global distribution spanning continents such as America, Asia, the Middle East, and Europe (He et al., 2022). These organisms are responsible for heart rot, a condition characterized by the growth within the central, non-living, woody tissues of standing trees. Notably, a single species of *Ganoderma* can target a multitude of host species, as observed in both temperate and tropical regions.

**Figure 8:**
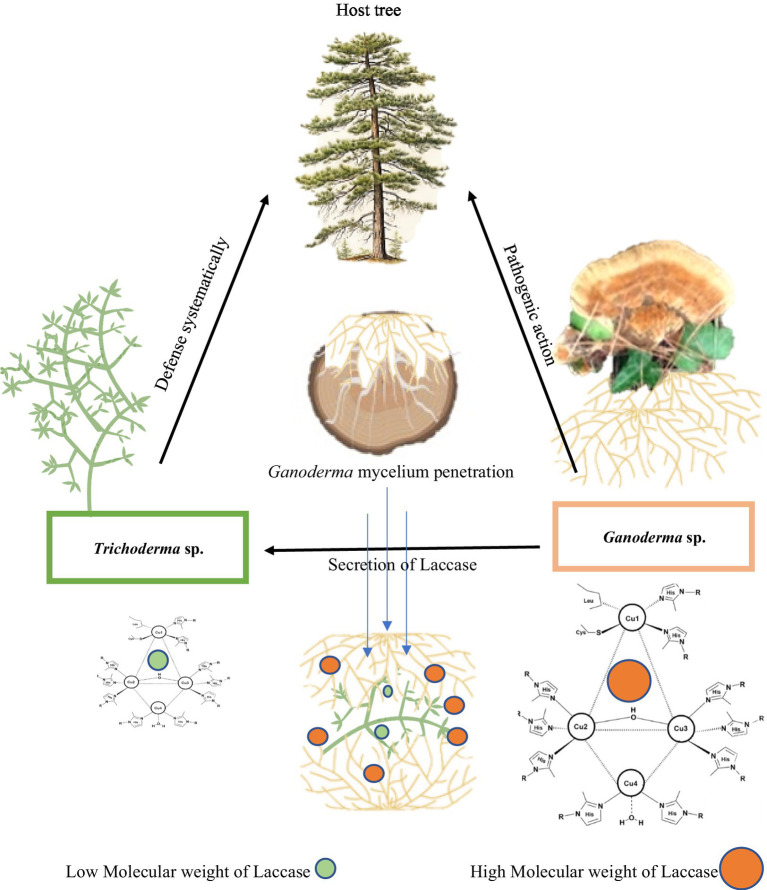
Mechanistic action of laccase of *Ganoderma camelum* against *Trichoderma atroviride.* Laccase of high molecular weight of *Ganoderma* antagonistically acts against the laccase of *Trichoderma* in competition of nutrients and space.

As biological control agents, pathogenic white fungi are frequently employed to manage plant and tree diseases. These fungi expedite wood degradation at a rate exceeding that of the pathogens. They vie for the same resources, compete for nutrients, synthesize inhibitory secondary metabolites, and possess the capacity to mycoparasitize the pathogens. Among other biological agents, *Trichoderma* competes with *Ganoderma* for wood resources by secreting laccase. Both species engage in a combative interaction, employing mycelial extension and laccase secretion to vie for space and nutrients (Figure 8).

The occurrence of brown spots on *Prosopis* wood is attributed to the action of *Ganoderma*, which facilitates the removal of carbohydrates, ultimately resulting in a brownish, oxidized lignin residue. The absence of a fibrous texture indicates the swift degradation of cellulose (Yang et al., 2022). As the infection progresses, the wood exhibits shrinkage upon drying, with cross-linings becoming discernible. Notably, non-enzymatic processes characterize the initial stages of infection. The fungal organism secretes a suite of enzymes that traverse the cell wall, analogous to a pair of scissors severing chains of hemicellulose and cellulose into minute fragments (Shankar et al., 2024).

Spores of *Ganoderma* penetrate trees through natural incisions, stomatal openings, or during pollination via ovules. Given the global biodiversity, approximately 300,000 plant species coexist with a multitude of “endophytic microbes,” including fungal species that inhabit palms across tropical and temperate zones (Dhillon et al., 2023). Notably, *Ganoderma boninense* is the causative agent of the devastating BSR disease affecting oil palms in Malaysia and Southeast Asia. This pathogen specifically targets palms aged of 4–5 years in replanted areas or regions with recurrent coconut palm cultivation (Supriyanto et al., 2024). The economic impact of BSR is estimated to range from 70 to 470 million dollars. Oil palm plantations situated on peatlands are particularly susceptible to BSR, with current control measures being limited to environmentally benign biological interventions, such as the utilization of *Trichoderma* (Sajjan et al., 2024). The potential of *Ganoderma*-antagonistic fungi to serve as a biological control agent for BSR in oil palms underscores the importance of exploring sustainable management strategies for this disease.

Mushroom diseases, growth inhibitors, environmental microorganisms, and pathogens pose significant challenges to the cultivation of *Ganoderma* (Ke et al., 2019; An et al., 2022). This study elucidates the competitive dynamics between *Trichoderma* spp. and *Ganoderma* during growth. In the cultivation of *Ganoderma*, the degradation of its culture substrate releases abundant exogenous carbon and nitrogen resources into the casing soil, creating a distinctive ecosystem (Carrasco et al., 2019; Cai and Druzhinina, 2021). In such environments, numerous *Trichoderma* species have been identified near *Ganoderma* habitats (Wang et al., 2016; Oh et al., 2018; Allaga et al., 2021). Characterized by their broad-spectrum antagonistic activity against microorganisms (López-Bucio et al., 2015), *Trichoderma* spp. demonstrate a competitive growth tendency against *Ganoderma* species. Observations indicate that *Trichoderma* spp. exert various effects on *G. lucidum*, *A. bisporus* (Innocenti et al., 2019), and *L. edodes* (Wang et al., 2016).

Antagonistic activities, including the production of antifungal metabolites and/or enzymes, mycoparasitism, and ecological competition (Vinale et al., 2008; Anees et al., 2010; Mukherjee et al., 2012), may significantly contribute to the proliferation of these organisms and potentially compromise the growth of *Ganoderma* species. Conversely, the broad perspective of microbial competition and antagonistic action in *Trichoderma* makes it one of the front-line microorganism employed to control the different plant pathogens (Zin and Badaluddin, 2020).

Direct systems involve space or nutrient competition, enzyme production, pathogen enzyme inactivation, and parasitism (Saragih et al., 2022). Studies have predominantly employed a solitary control agent against a singular pathogen in the management of plant pathogens. Nevertheless, given the diverse environmental demands of various microorganisms, this strategy may not be universally effective across all the soil types. Moreover, naturally occurring biological control agents are more likely to function as mixed antagonistic communities than individual antagonist. These antagonistic blends exhibit greater stability and a broader spectrum of activity, thereby augmenting the dependability and effectiveness of biological control (Ongena and Jacques, 2008). Numerous antagonistic species, such as *Trichoderma* (Susanto et al., 2005), have effectively controlled *Ganoderma* colonization.

Symbiotic associations with host plants facilitate the mitigation of diverse stress forms, enhancement of plant metabolism, and increased biomass by various *Trichoderma* species (Susanto et al., 2005). A study by Go et al. (2019) elucidated that mycelial interaction is a primary method for evaluating the antagonistic efficacy of potent bioagents against fungal pathogens.

The antagonistic activity varied depending on the used medium (Saragih et al., 2022). Similar findings were observed by Mustafa et al. (2019), who indicated that PDA is the best medium for the growth of *Trichoderma* sp. and the performance of antagonistic activity. Naher et al. (2015) documented the antagonistic mechanism of *Trichoderma* sp. against the pathogen of *G. boninense*, wherein *Trichoderma* sp. entwined around the hyphae of *G. boninense* to counteract the pathogen.

Nascimento Brito et al. (2023) isolated three *Trichoderma* species from cocoa and rubber plants to counteract diseases caused by *G. boninense*. Culture experiments revealed that *T. harzianum* markedly inhibited the growth of *G. boninense*, with inhibition rates ranging from 47.86% (9 days) to 72.06% (14 days) (Rubio et al., 2017). The formation of an inhibition zone by *Ganoderma* species was visibly observed in this study.

The antagonistic impact of *Phlebiopsis gigantea* against *Heterobasidion annosum*, a root/butt rot pathogen affecting conifers, has been documented (Bruna et al., 2020). Similarly, *Pycnoporus sanguineus*, *Grammothele fuligo*, and *Trametes lactinea*, which naturally occur on oil palm trunks, have also been identified as possessing antagonistic activity against *G. boninense* (Naidu et al., 2015).

Endophytic pathogenic basidiomycetes have been investigated for their efficacy in combating fungal diseases in cacao (Rodríguez Velázquez et al., 2024). *Schizophyllum commune* and *T. lactinea* have demonstrated potential in controlling endophytic basidiomycetes (*G. boninense*) within oil palm plantations. Asymptomatic endophytic basidiomycetes can interfere with the activities of pathogens occupying the same ecological niche, as evidenced by the interaction between *P. gigantea* and *H. annosum* in conifers (Rodríguez Velázquez et al., 2024).

In conclusion, this study offers comprehensive insights into the identification of new *Ganoderma* species. The study highlights the potential challenges posed by laccases of *Trichoderma* species to *Ganoderma* species and also demonstrates the antagonistic dynamics between these fungi. To the best of our knowledge, this research presents novel data on the newly identified *Ganoderma* species and its pathogenic impact on host trees.

Continuous co-evolution within fungal pathogens results in the emergence of new races annually. *Trichoderma* species engage in complex interactions with other fungal species to address these evolving races. However, a profound understanding of the ecology of *Ganoderma* species is crucial for effectively managing the fungal diseases. Biotechnology holds promising potential for enhancing *Trichoderma* efficacy in controlling fungal pathogens and elucidating pathogenic mechanisms. The extensive use of fungicides has led to the proliferation of *Trichoderma* species to combat emerging pathogen races. Yet, the efficacy of individual applications is challenging to analyze. The application of any microbial agent necessitates careful consideration of technical performance and the safety of human health.

Moreover, *Ganoderma* species can be integrated with conventional chemicals for more rapid and effective disease management. The successful application of both species relies heavily on the contributions from research and developmental efforts across various industries, as well as support from governments, and non-governmental organizations.

Ultimately, while *Trichoderma* species have not completely eradicated the newly identified *Ganoderma* species, they have significantly reduced disease incidence with minimal crop damage. *Trichoderma* plays a pivotal role in managing plant-associated pathogens, predominantly soil-borne fungal species, but it remains ineffective against the pathogenicity of *Ganoderma* species. Despite of extensive research on *Trichoderma*, further investigation into its utilization and its interaction with the newly identified *Ganoderma* species is warranted.

## Data Availability

The original contributions presented in the study are included in the article/Supplementary material, further inquiries can be directed to the corresponding authors.
