# Supplementary figures and images for: Competitive antagonistic action of laccase between Trichoderma species and the newly identified wood pathogenic Ganoderma camelum
Source: Front Microbiol. 2024 Sep 25;15:1408521. doi: 10.3389/fmicb.2024.1408521 (PMC11461316; doi:10.3389/fmicb.2024.1408521)

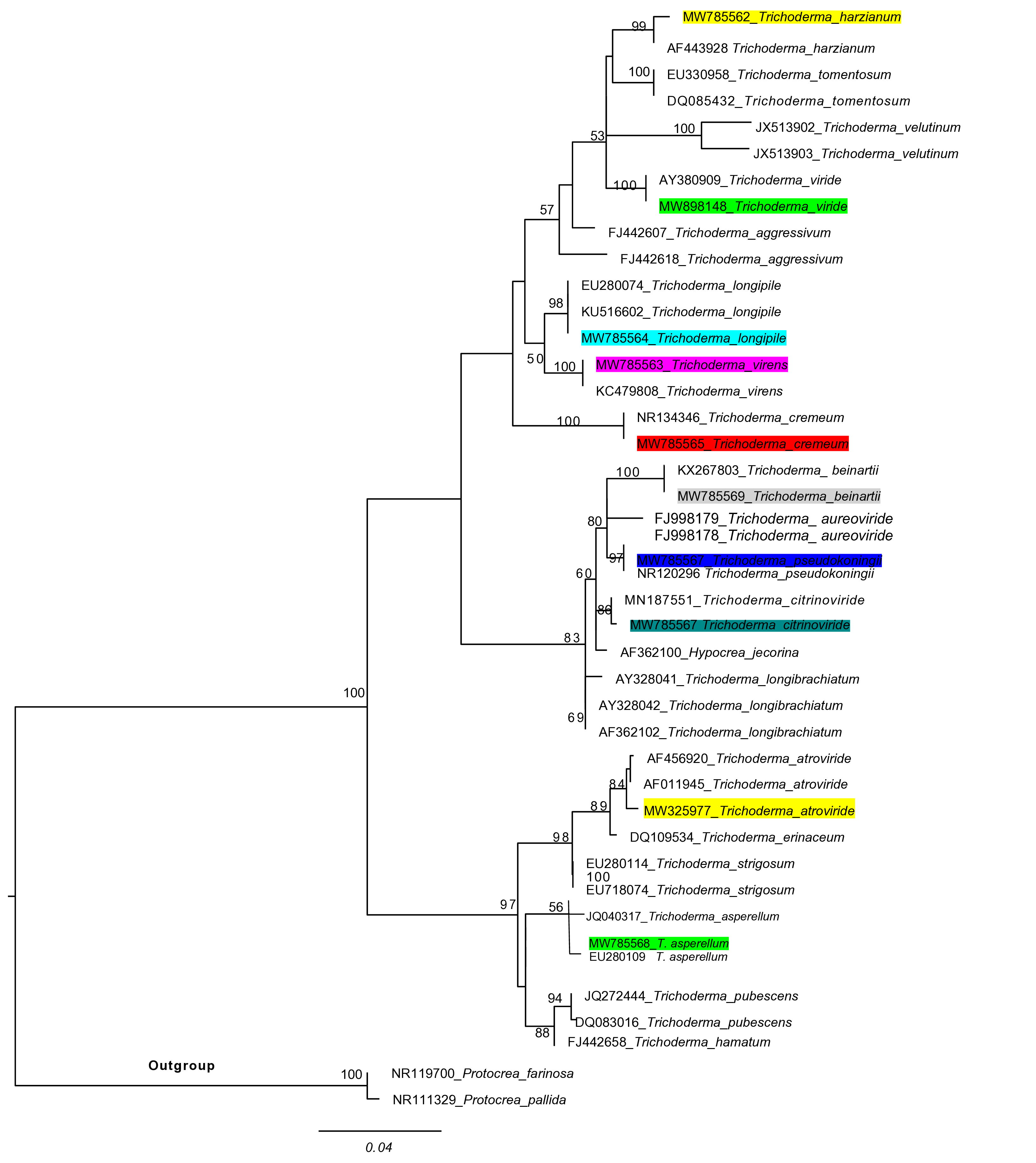

Supplement: Supplementary file 2 [file Image_1.JPEG]

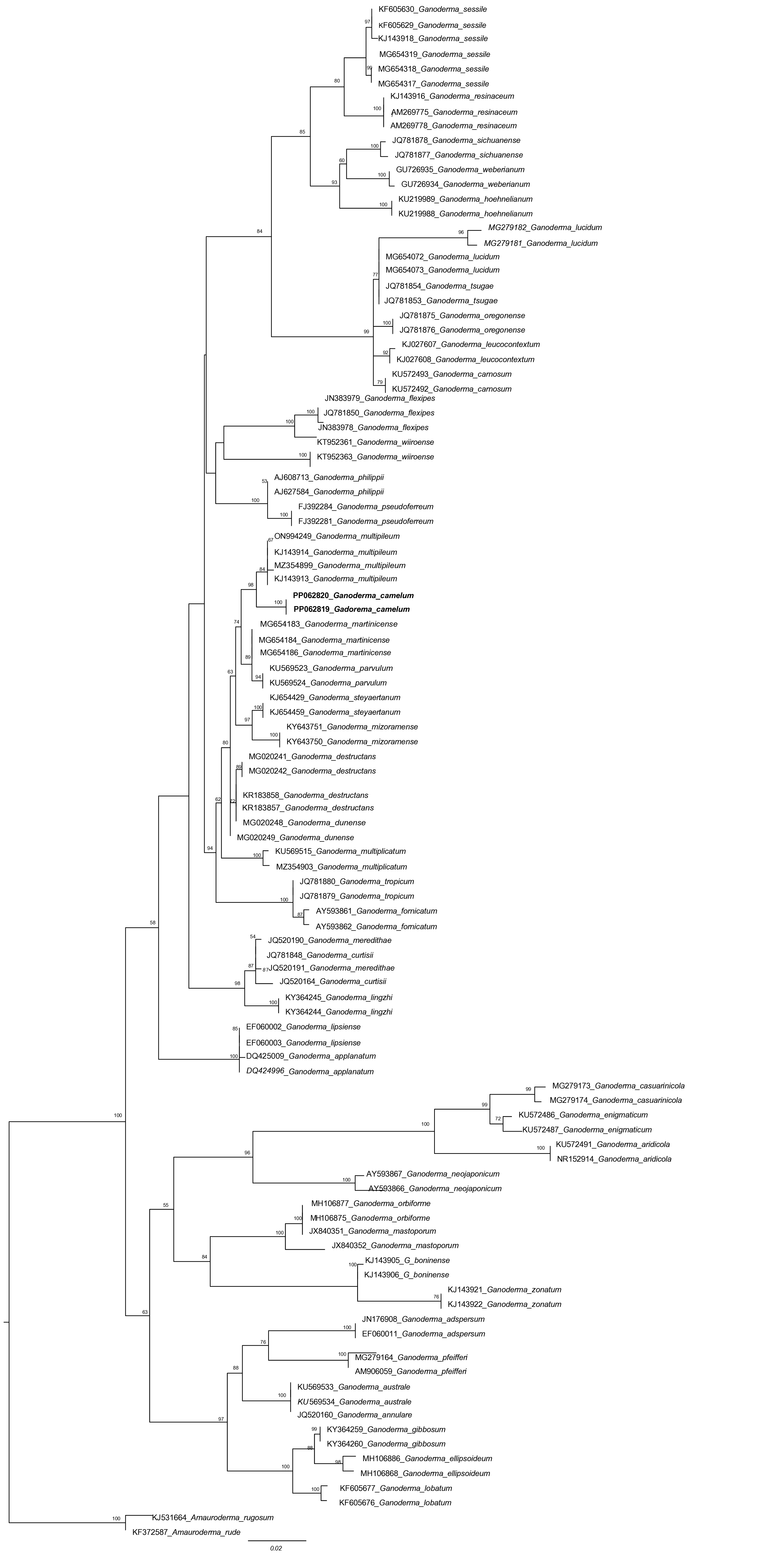

Supplement: Supplementary file 3 [file Image_2.JPEG]
