# Supplementary material for: Competitive antagonistic action of laccase between Trichoderma species and the newly identified wood pathogenic Ganoderma camelum
Source: Front Microbiol. 2024 Sep 25;15:1408521. doi: 10.3389/fmicb.2024.1408521 (PMC11461316; doi:10.3389/fmicb.2024.1408521)
Supplement: Supplementary file 4 [file Presentation_1.pdf]

Competitive antagonistic action of laccase between *Trichoderma* species and the newly identified wood pathogenic *Ganoderma camelum*

Aisha Umar[1,2*], Mohamed S Elshikh[3], Reem M. Aljowaie[3], Juma Mahmud Hussein[4], Laurent Dufossé[5], Chenghong Wu[1], Junxing Lu[1*]

[1]Chongqing Key Laboratory of Plant Environmental Adaptations, College of life science, Chongqing Normal University, Chongqing, China.
[2]Institute of Botany, University of the Punjab, Lahore, Pakistan.
[3]Department of Botany and Microbiology, College of Science, King Saud University, Riyadh, Saudi Arabia.
[4] Department of Molecular Biology and Biotechnology, University of Dar es Salaam, Dar esSalaam, Tanzania
[5] CHEMBIOPRO Laboratoire de Chimie et Biotechnologie des Produits Naturels, ESIROI Agroalimentaire, Université de La Réunion, 15 Avenue René Cassin, F-97400 Saint-Denis, Ile de La Réunion, France.

*Correspondence: ash.dr88@gmail.com (AU); junxlu@163.com(JL)

List of Abbreviations:

ITS = Internal Transcribed Spacer

U/mL= Enzyme Activity Unit/mL

E.A= Enzyme Activity

CTAB= Cetyl trimethylammonium bromide

MAFFT= Multiple Alignment using Fast Fourier Transform

MEGA= Molecular Evolutionary Genetics Analysis

PIRG= percentage inhibition of radial growth

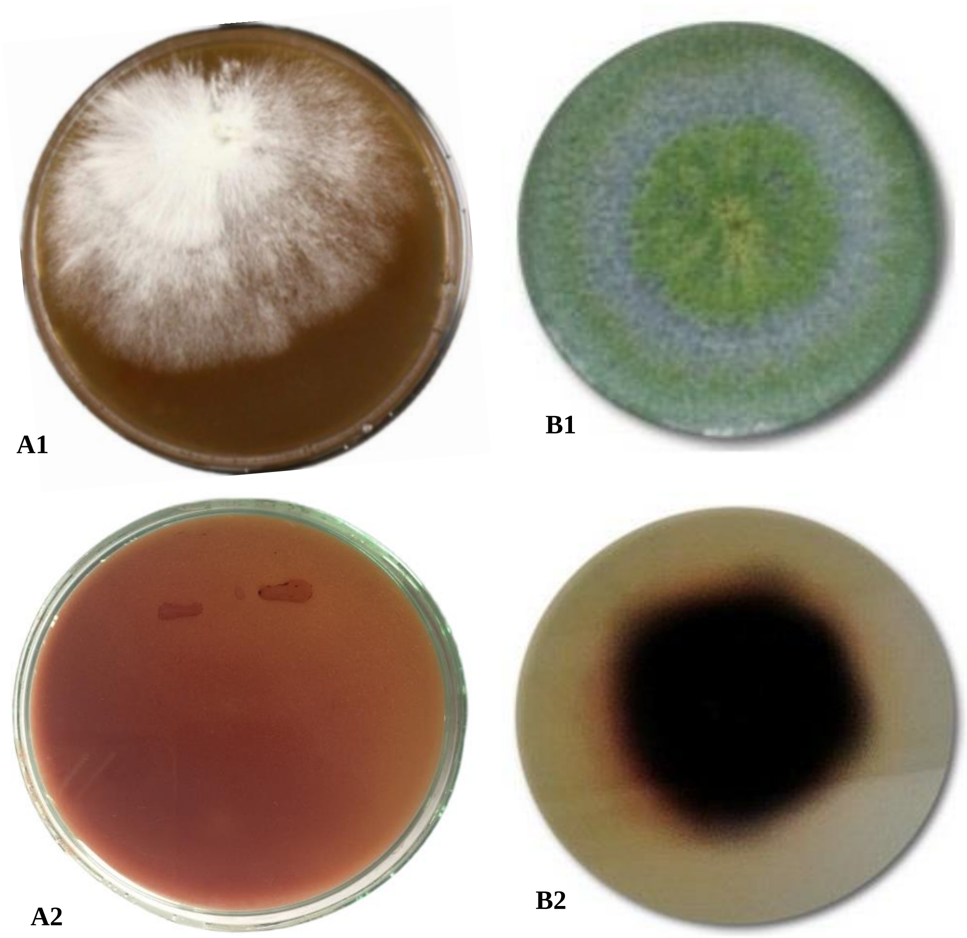

Figure S1. A1: Pure cultural mycelium of *Ganoderma camelum* sp. nov., **A2:** Guaiacol oxidation by laccase of *G. camelum*, **B1:** Pure cultural mycelium of *Trichoderma atroviride*, **B2:** Guaiacol oxidation by laccase of *T. atroviride.*

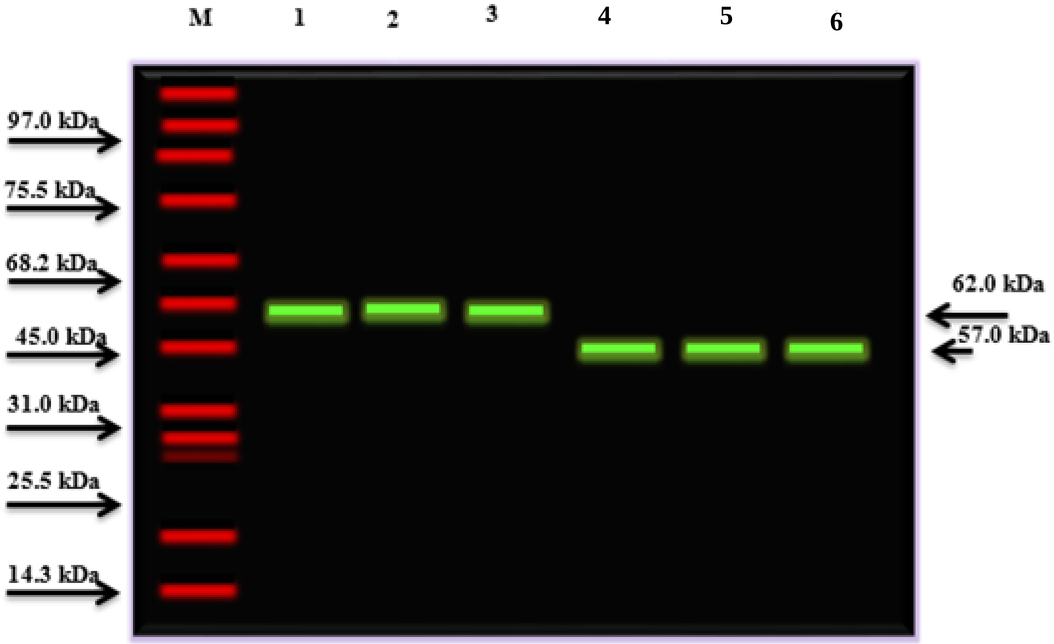

Figure S2. SDS–PAGE analysis of *G. camelum* (Line 1,2,3) and *T. atroviride* (Line 4,5,6), Molecular protein weight markers (M).

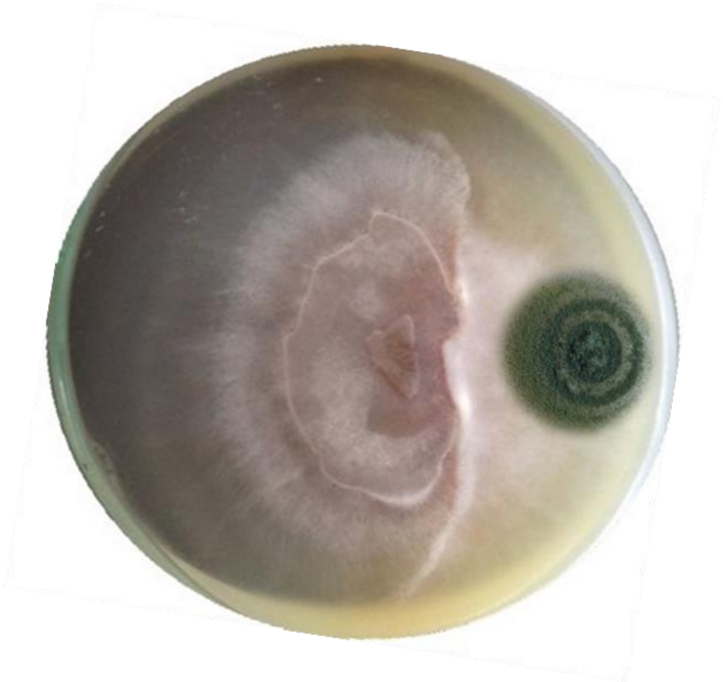

Figure S3. Antagonistic mycelium confrontation of *G. camelum* towards *T. atroviride.*

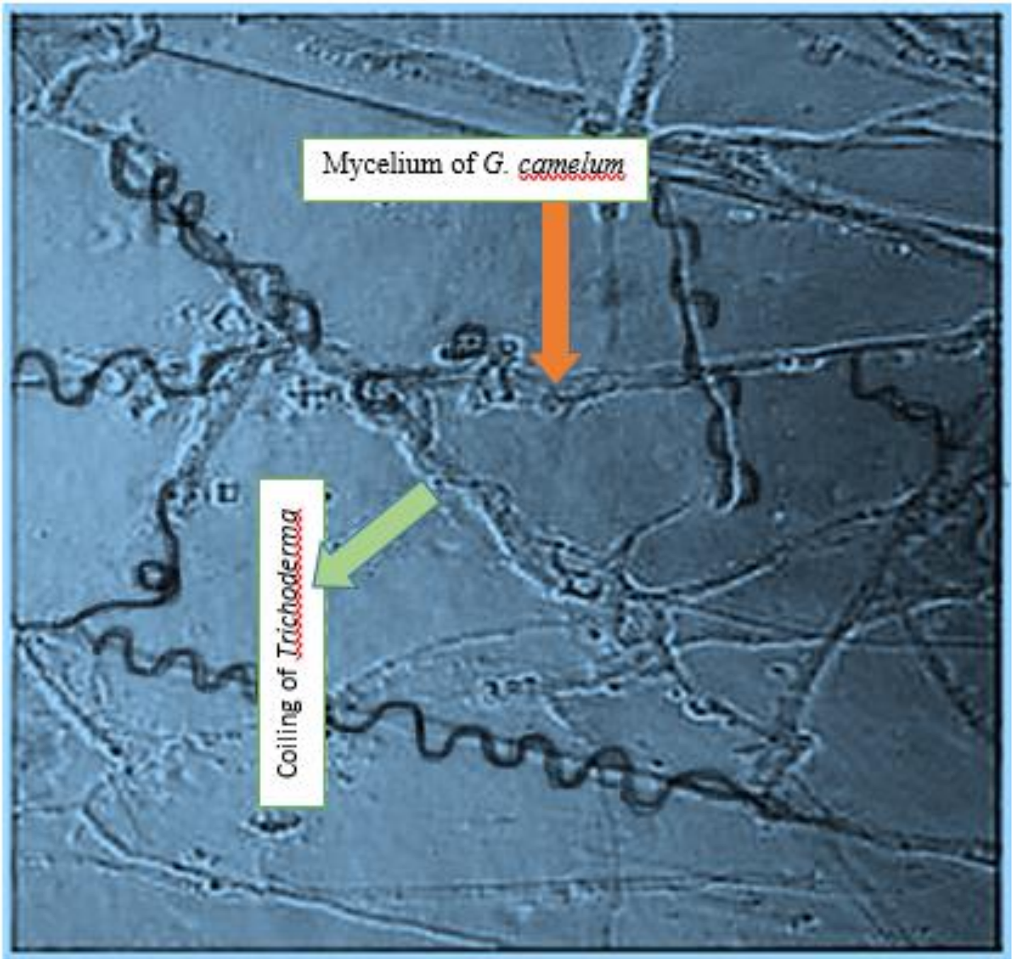

Figure S4. Microscopic anatomical abnormalities in the mycelium and hyphal structure *G. camelum* and *T. atroviride*.
